# Low-dose naltrexone extends healthspan and lifespan in *C. elegans* via SKN-1 activation

**DOI:** 10.1016/j.isci.2024.109949

**Published:** 2024-05-08

**Authors:** Weisha Li, Rebecca L. McIntyre, Bauke V. Schomakers, Rashmi Kamble, Anne H.G. Luesink, Michel van Weeghel, Riekelt H. Houtkooper, Arwen W. Gao, Georges E. Janssens

**Affiliations:** 1 Laboratory Genetic Metabolic Diseases, Amsterdam UMC, University of Amsterdam, Amsterdam, the Netherlands; 2 Amsterdam Gastroenterology, Endocrinology and Metabolism Institute, Amsterdam UMC, University of Amsterdam, Amsterdam, the Netherlands; 3 Core Facility Metabolomics, Amsterdam UMC Location University of Amsterdam, 1105 AZ Amsterdam, the Netherlands; 4 Amsterdam Cardiovascular Sciences, Amsterdam, the Netherlands

**Keywords:** Biological process, Cellular physiology, Molecular biology

## Abstract

As the global aging population rises, finding effective interventions to improve aging health is crucial. Drug repurposing, utilizing existing drugs for new purposes, presents a promising strategy for rapid implementation. We explored naltrexone from the Library of Integrated Network-based Cellular Signatures (LINCS) based on several selection criteria. Low-dose naltrexone (LDN) has gained attention for treating various diseases, yet its impact on longevity remains underexplored. Our study on *C. elegans* demonstrated that a low dose, but not high dose, of naltrexone extended the healthspan and lifespan. This effect was mediated through SKN-1 (NRF2 in mammals) signaling, influencing innate immune gene expression and upregulating oxidative stress responses. With LDN’s low side effects profile, our findings underscore its potential as a geroprotector, suggesting further exploration for promoting healthy aging in humans is warranted.

## Highlights

• Low-dose naltrexone (LDN) extends the healthspan and lifespan in *C. elegans*
• LDN promotes the nuclear translocation of transcription factor SKN-1
• Lifespan and healthspan extension of LDN depend on SKN-1
• LDN activates the oxidative stress response and certain immunity genes

## Graphical abstract

**Figure:**
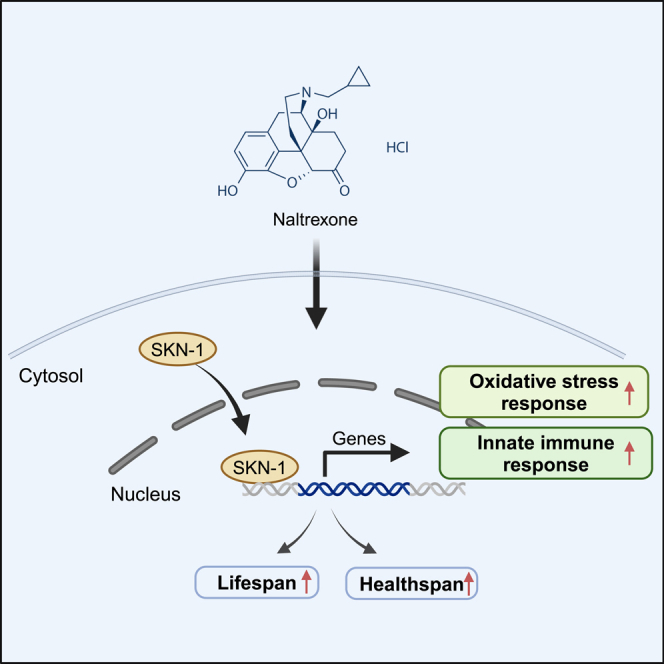

## Introduction

Many countries are facing a significant challenge in dealing with the unprecedented and rapid pace of population aging. The World Health Organization’s report reveals that the number of individuals aged 60 years and above was 1 billion in 2019.^1^^,^^2^ Aging is a primary risk factor for various diseases and conditions.^3^ It is therefore necessary for a concerted effort to reduce health disparities and enhance the quality of life for older individuals by promoting healthy aging.

There is increasing interest in the concept of aging as a druggable target to prevent age-related diseases. However, developing new drugs to address human aging presents challenges in conducting clinical trials. In the absence of validated risk biomarkers, a large and initially healthy population would need to be treated over an extended period, making it difficult to conduct trials.^4^ Therefore, repurposing existing drugs with a good safety profile is a more practical short-term solution than developing new drugs. For instance, the use of the computational screening of drugs with publicly available databases, such as the Library of Integrated Network-based Cellular Signatures (LINCS), provides simple, important platforms available to describe gene expression “signatures” stimulated by small molecules in cell lines.^5^^,^^6^^,^^7^ Genetic approaches have effectively prolonged the lifespan of model organisms.^8^ These investigations have uncovered conserved genes and pathways that play a crucial role in regulating longevity and promoting healthy aging. Consequently, geroprotective drugs that specifically target these pathways can be identified.^9^

Naltrexone is a prescription medication approved by the US Food and Drug Administration (FDA) in 1984 for the treatment of alcohol use disorder and opioid use disorder.^10^ It belongs to a class of drugs called opioid antagonists.^11^ In recent years, there have been several significant findings regarding a specific dosage of naltrexone called low-dose naltrexone (LDN). LDN has been shown to have immune-modulating properties that could reduce various oncogenic and inflammatory autoimmune processes and alleviate symptoms of certain mental ailments.^12^^,^^13^^,^^14^^,^^15^ These studies have demonstrated its potential in promoting general health sustainability. As such, naltrexone may have broader therapeutic applications beyond addiction medicine, with greater unexplored potential.

Here, we studied the potential benefits of low-dose naltrexone (LDN) in promoting healthy aging using *Caenorhabditis elegans* as a model organism. We found that LDN treatment extended both healthspan and lifespan in worms, while high-dose naltrexone did not produce the same effects. Further metabolomics analysis revealed that LDN treatment induced metabolic changes that led to increased activity of both amino acid and glucose metabolism, but the longevity effect was independent of the DAF-16*/*FOXO3 signaling. We then tested various mutant strains and found that the lifespan extension induced by LDN treatment was dependent on the SKN-1/NRF2 transcription factor. We also observed that LDN treatment not only increased the expression of innate immune genes but also upregulated the oxidative stress response, in line with a role for SKN-1/NRF2 in LDN’s lifespan promoting effects. Inhibiting the nuclear translocation of SKN-1 from the cytosol could attenuate the LDN-mediated innate immune gene expression and oxidative stress response. Overall, our study highlights the potential of LDN as a therapeutic agent for promoting healthy aging and identifies its mechanism of action.

## Results

### Identification of naltrexone as a geroprotective compound

**Figure 1:**
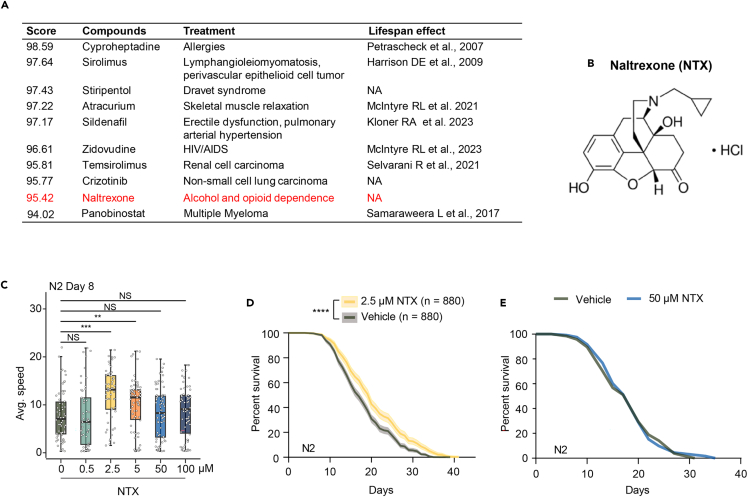
Low-dose naltrexone (LDN) promotes healthspan and lifespan in *C. elegans* (A) List of the top 10 FDA-approved drugs that have similar effects at the transcriptional level as observed in FOXO3 overexpression cells, retrieved from the LINCS database. (B) Molecular structure of opioid antagonist naltrexone (NTX). (C) Mobility of N2 worms upon feeding with either water or various doses of NTX, measured at day 8 of adulthood. NTX promotes healthspan in a dose-dependent manner in wild-type (N2) worms. Significance was determined using One-way ANOVA. (D) NTX extends worm lifespan at 2.5 μM. *N* = 880 worms, a lifespan composite of 8 independent lifespan assays (see Figure S1). (E) There is no effect on the lifespan when treating worms with 50 μM NTX. *N* = 120 worms. The statistical analysis of the survival curves is performed using log rank test. ∗∗∗∗*p* < 0.0001, ∗∗∗*p* < 0.001, ∗∗*p* < 0.01, ∗*p* < 0.05, NS: not significant.

To identify potential geroprotective compounds, we previously screened for drugs that could mimic the overexpression of the longevity transcription factor FOXO3 (*daf-16* in worms).^16^ Pharmaceutical modulation of the FOXO3 signaling pathway has emerged as a promising avenue for promoting healthy longevity.^17^^,^^18^ This led us to identify the longevity effects of atracurium, which extends lifespan in *C. elegans* through the activation of the DAF-16/FOXO transcription factor,^16^ and zidovudine, which extends lifespan independently of DAF-16/FOXO, acting rather through the ATF4 longevity transcription factor.^19^ In order to expand the range of compounds that can mimic FOXO3 overexpression, we re-analyzed the ranked compounds list obtained from the LINCs dataset used in our initial screening for FOXO3 overexpression gerorpotectors.^16^^,^^19^ We focused on repurposing existing drugs, and therefore non-FDA-approved compounds on the table were excluded (Figure 1A). This list included atracurium and zidovudine, as previously described,^16^^,^^19^ in addition to other compounds that either extend lifespan in model organisms directly, reduce senescence phenotypes, or are associated with lower mortality in humans. For example, cyproheptadine, sirolimus (also known as rapamycin), and temsirolimus have exhibited significant geroprotective properties beyond their current clinical applications.^20^^,^^21^^,^^22^^,^^23^^,^^24^ Panobinostat has an anti-senescence role in chemotherapy-treated cancer cells.^25^ PDE5 inhibitors, such as sildenafil, have been associated with lower mortality rates in humans.^26^

Here, we chose to focus our study on naltrexone (NTX), which (a) has been unexplored mechanistically in relation to longevity effects, (b) is highly ranked in our screen, and (c) may possess low side effect profiles for a greater probability of translation to humans. NTX was used as an opioid antagonist and approved by the FDA in 1985 to treat opiate dependence^10^ (Figure 1B). To evaluate the effects of NTX on healthspan and lifespan, we turned to *C. elegans* worms, a well-accepted model for aging research. We assayed healthspan—which we defined as the crawling speed of the worms—at 8 days of adulthood. This timepoint has been shown to be reliable for assessing healthspan in aging in worms by us^16^ and others.^27^^,^^28^ We found that lower doses of NTX (2.5 μM and 5 μM) were effective in extending healthspan and lifespan in wild-type (N2) worms. Interestingly, higher doses (50 μM and 100 μM) had no significant impact (Figure 1C). Similarly, low-dose NTX (2.5 μM) extended worm lifespan with a 17.6% increase in median lifespan relative to control-fed, while high-dose NTX (50 μM) did not (Figures 1D, 1E, and S1A–S1H; Table S1). Overall, our findings suggest that low doses of NTX are a lifespan-extending agent in *C. elegans*, whereas higher doses do not confer these benefits.

### Metabolomics profiling reveals a metabolic rewiring in worms upon low-dose naltrexone

**Figure 2:**
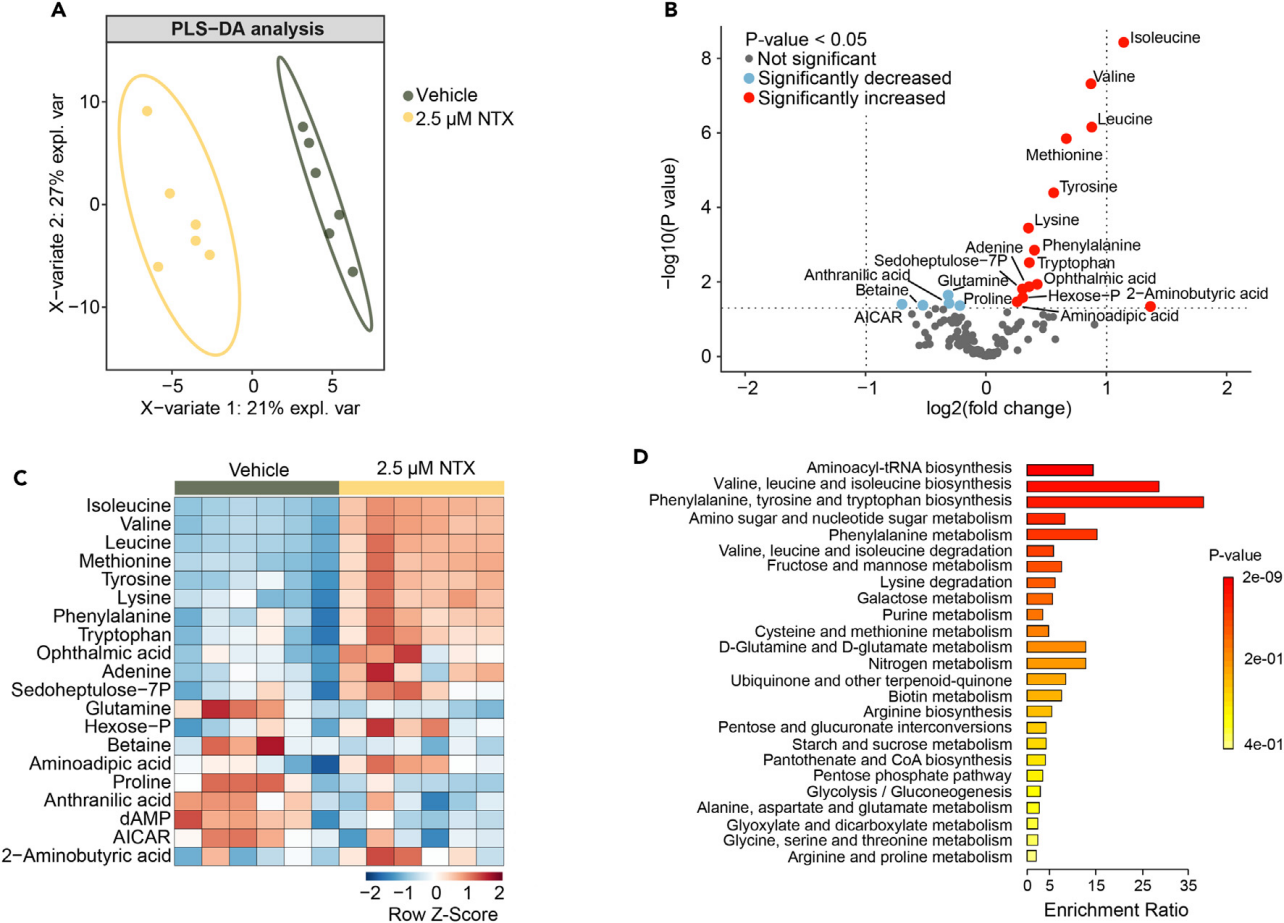
Metabolomics profiling reveals a metabolic rewiring in worms upon LDN (A) Partial least squares discriminant analysis (PLS-DA) showing group separation on the basis of the metabolite profiles in day 3 adult worms treated with NTX (2.5 μM) compared with control worms (*n* = 6 biological samples per condition). (B) Differential analysis showing significantly altered metabolites (*p* < 0.05). Statistical testing of differential expression was performed using empirical Student’s t test. (C) The top 20 altered metabolites ranked according to their variable of importance (VIP) derived from the PLS-DA. (D) Metabolite Set Enrichment Analysis (MSEA) shows the most altered metabolic pathways in NTX-treated worms compared with control worms. MSEA was analyzed using online tools (MetaboAnalyst 3.0 software). Color intensity (yellow to red) reflects increasing statistical significance. Metabolomics datasets can be found in Table S2.

To investigate the metabolic effects of LDN and its role in lifespan extension, we collected worms treated with LDN on day 3 of adulthood and performed metabolomics analysis using UPLC-mass spectrometry (Table S2). We found a clear separation between the two treatment groups by using partial least squares discriminant analysis (PLS-DA), suggesting a distinct effect of the LDN treatment (Figure 2A), consisting of 14 metabolites significantly increased and five significantly decreased in abundance (*p*-value <0.05) (Figure 2B). Among these metabolic changes, the most striking was the accumulation of amino acids (Figure 2C). Indeed, metabolite set enrichment analysis (MSEA) confirmed that the most altered metabolic pathways in LDN-treated worms were related to amino acid metabolism (Figure 2D).

In addition to supporting protein synthesis, amino acids also control immune cell function, playing a key role in regulating different steps of innate immunity, which has been previously shown to be of interest regarding LDN’s effects.^29^^,^^30^ The amino acids that were increased after LDN treatment included isoleucine, valine, leucine, methionine, tyrosine, lysine, phenylalanine, and tryptophan (Figure 2B). In the top altered metabolites, we also found that LDN treatment resulted in high levels of ophthalmic acid, an oxidative stress marker (Figure 2C).^31^^,^^32^^,^^33^ In line with this, we found pathways enriched related to glucose metabolisms in the MSEA, such as amino sugar and nucleotide sugar metabolism, fructose and mannose metabolism and galactose metabolism (Figure 2D). Oxidative stress significantly impacts glucose homeostasis regulation, suggesting a possible link^34^^,^^35^ (Figure 2D). Together, these metabolic changes pointed to an age-related change in oxidative stress response and immune system related changes in LDN treated worms.

### Low-dose naltrexone improves immune gene expression and oxidative stress responses and extends lifespan independent of *daf-16* signaling

**Figure 3:**
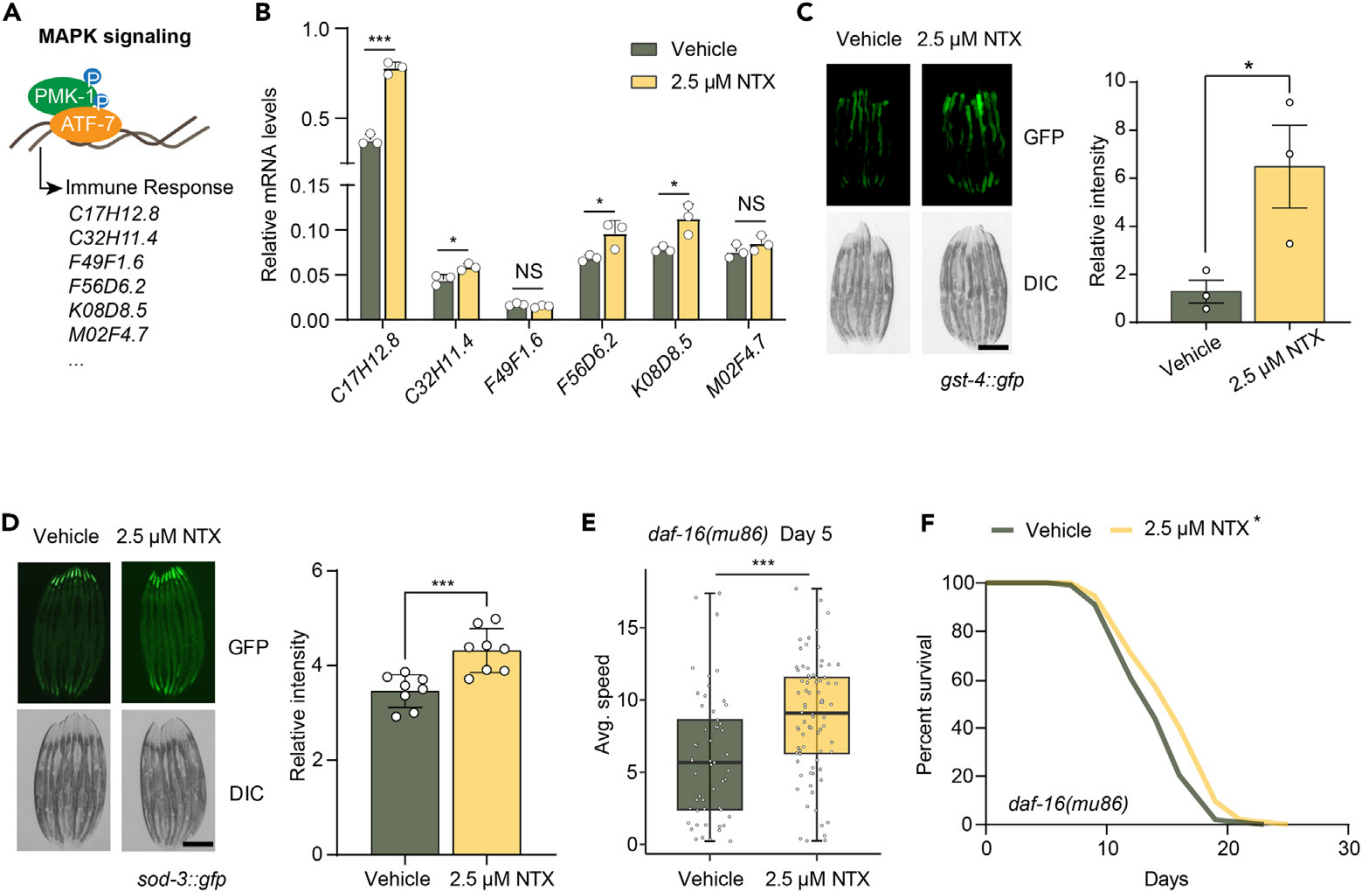
LDN improves innate immune gene expression and oxidative stress responses and extends worm lifespan independent of *daf-16* signaling (A) Pathway for the activation of p38MAPK. (B) The mRNA level of innate immune genes in 2.5 μM NTX-treated worms was determined by qRT-PCR. Bar graphs indicate mean ± SD. Significance was determined by a Student’s t test. Reference genes: *pmp-3, cdc-42*. (C) Representative fluorescence microscopy images and quantification of day 3 adult worms showing GST-4:GFP expression treated with or without 2.5 μM NTX. Scale bar: 0.3 mm. (D) Representative fluorescence microscopy images and quantification of SOD-3:GFP fluorescence treated with or without 2.5 μM NTX for 3 days. Scale bar: 0.3 mm. (E) Mobility of *daf-16(mu86)* worms treated with or without 2.5 μM of NTX at day 5 of adulthood. Significance was determined using Student’s t test. (F) Survival of *daf-16(mu86)* worms treated with or without 2.5 μM NTX. *N* = 120 worms. The statistical analysis of the survival curves was performed by log rank test. ∗∗∗*p* < 0.001, ∗∗*p* < 0.01, ∗*p* < 0.05, NS: not significant.

Previous studies have noted the immunoregulatory and oxidative damage prevention effects of naltrexone, though no clear mechanism has been proposed.^15^^,^^36^^,^^37^ To investigate the specific functional influences of LDN treatment, we evaluated the expression of widely studied p38MAPK/ATF-7-dependent innate immune genes in worms and observed an increase in these genes upon NTX treatment, including increases in *C17H12.8, C32H11.4, F56D6.2* and *K08D8.5* gene expression^38^^,^^39^^,^^40^ (Figures 3A and 3B). Furthermore, to determine whether the antioxidant genes *sod-3* and *gst-4* play a role in LDN treatment, we examined their expression through the use of GFP reporter strains (*gst-4::GFP* and *sod-3::GFP* strains).^41^ LDN increased the GFP signal of *sod-3* and *gst-4* (Figures 3C and 3D). Given that the compounds list was screened based on mimicking FOXO3 overexpression, we sought to determine whether the lifespan extension induced by LDN was dependent on *daf-16* (the worm gene orthologous to the FOXO genes in mammals). We therefore assessed the mobility of *daf-16(mu86)* worms to determine their healthspan. Considering the shorter lifespan of *daf-16* mutants, we assessed healthspan on day 5 (rather than day 8 as in N2) and we observed a significant increase in the healthspan of LDN-treated *daf-16(mu86)* worms (Figure 3E). This finding suggests that the effects of LDN on healthspan are independent of DAF-16/FOXO signaling. We subsequently evaluated whether *daf-16* was required for LDN-induced lifespan extension, and tested the effects of LDN in worms possessing a mutation in the DAF-16/FOXO gene. The results revealed that LDN still extended lifespan in the *daf-16(mu86)* mutants (Figure 3F; Table S1). These findings confirmed that LDN’s healthspan and lifespan extension effects were not dependent on *daf-16*, thus emphasizing the need for further investigation to better understand the underlying pathways involved.

### Low-dose naltrexone extends lifespan in *C. elegans* dependent on SKN-1/NRF2

**Figure 4:**
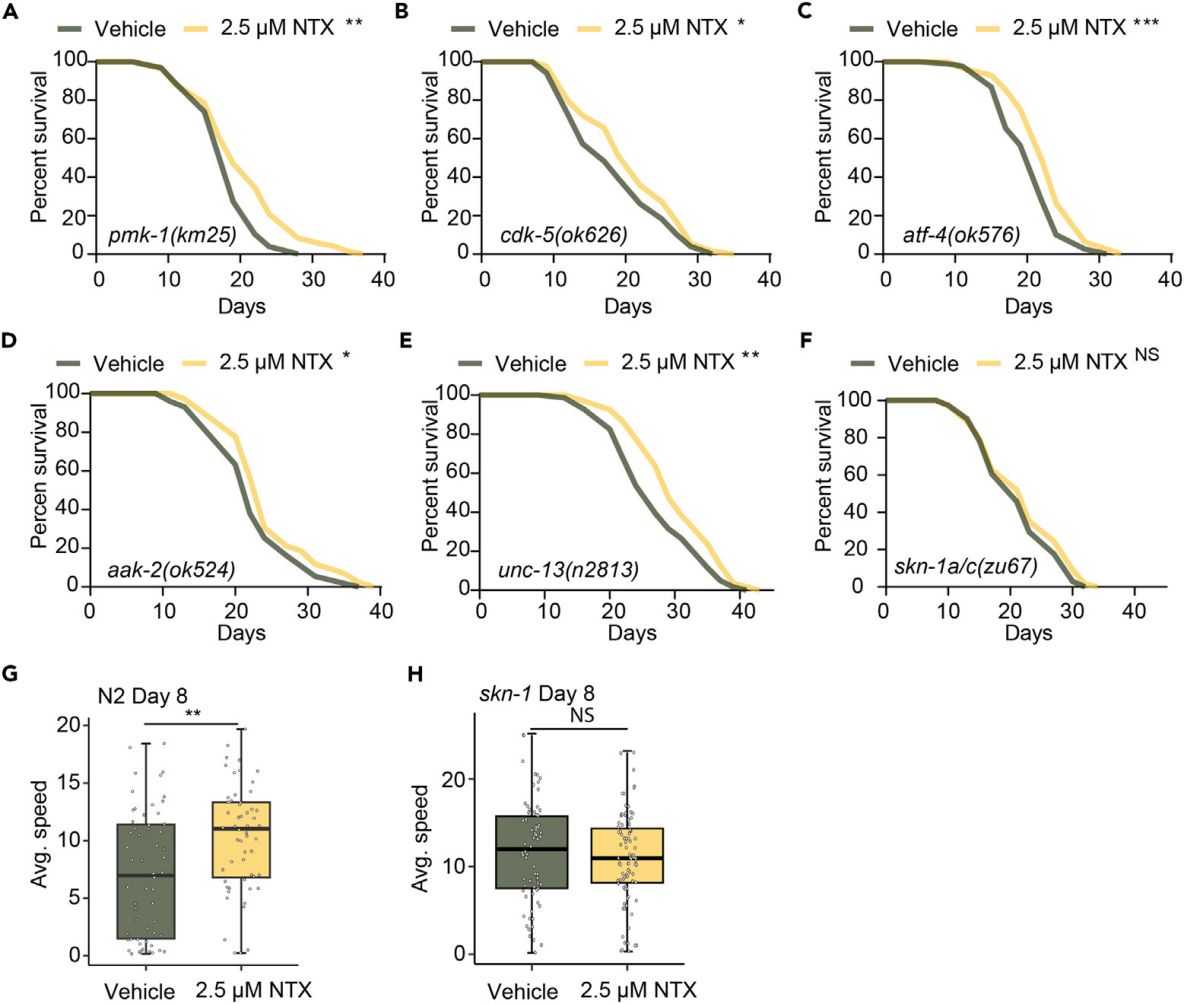
LDN-mediated lifespan extension is dependent on SKN-1 signaling (A–F) Survival of *pmk-1(km25)* (A), *cdk-5(ok626)* (B), *atf-4(ok576)* (C), *aak-2(ok524)* (D), *unc13(n2813)* (E), *skn-1(zu67)* (F) worms treated with control and 2.5 μM NTX. *N* = 100 worms. The statistical analysis is performed by the log rank test. (G) Mobility testing at day 8 of adulthood of N2 worms upon feeding with either water vehicle or 2.5 μM NTX. Avg. speed = average speed. Significance was determined using the Student’s t test. (H) Mobility of *skn-1(zu67)* worms treated with 2.5 μM of NTX at day 8 of adulthood. Significance was determined using the Student’s t test. ∗∗∗*p* < 0.001, ∗∗*p* < 0.01, ∗*p* < 0.05, NS: not significant.

**Figure 5:**
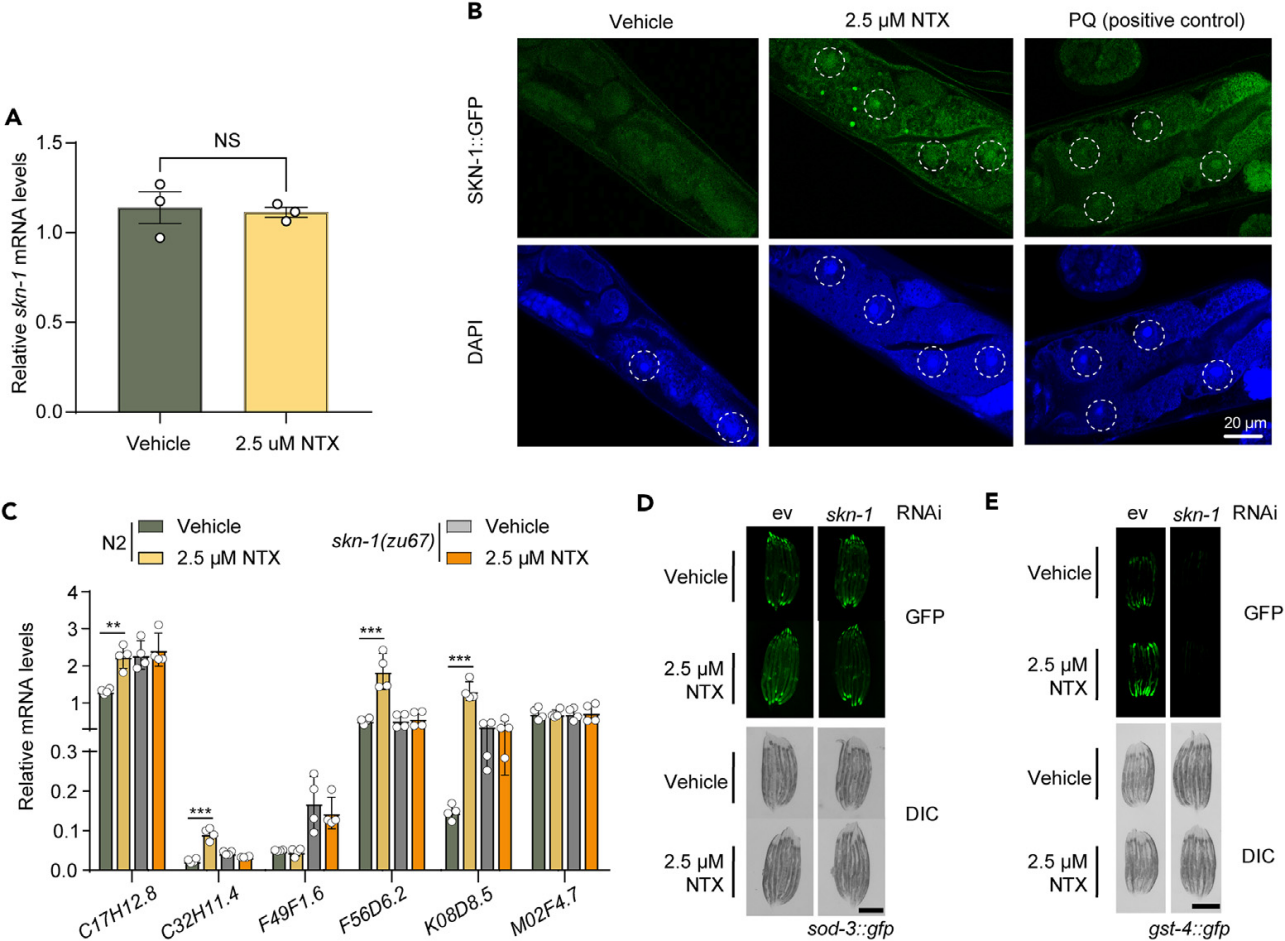
LDN promotes the translocation of transcription factor SKN-1 from the cytosol to the nucleus and activates the oxidative stress response (A) mRNA level of *skn-1* gene in control worms and worms treated with 2.5 μM NTX, determined by qRT-PCR. Expression of *skn-1* does not change upon NTX treatment. Bar graphs indicate mean ± SD. Significance was determined by an unpaired Student’s t test. (B) Representative images of day 3 adult worms showing the translocation of the transcription factor SKN-1 from cytosol to the nucleus in worms fed with or without 2.5 μM NTX. Postive control: 100mM paraquat (PQ). (C) The mRNA level of innate immune genes in either N2 or *skn-1(zu67)* worms treated with or without 2.5 μM NTX, determined by qRT-PCR. Reference gene: pmp-3, rbd-1. Bar graphs indicate mean ± SD. Significance was determined by a One-way ANOVA between vehicle and NTX treatment comparisons. (D) Representative images of day 3 adult worms (stage-matched) showing SOD-3:GFP expression in N2 or *skn-1(zu67)* worms fed with or without 2.5 μM NTX. Scale bar: 0.3 mm. (E) Representative images of day 3 adult worms (stage-matched) showing GST-4:GFP expression in N2 or *skn-1(zu67)* worms fed with or without 2.5 μM NTX. Scale bar: 0.3 mm. ∗∗∗*p* < 0.001, ∗∗*p* < 0.01, ∗*p* < 0.05, NS: not significant.

We next aimed to uncover the mechanism allowing for LDN’s lifespan and healthspan benefits. To do so, we prepared a small-scale screen, testing the effects of LDN in worms deficient in various pathways emerging from our analyses. We tested the innate immune response (*pmk-1* mutant), the oxidative stress response (*skn-1* mutant),^42^^,^^43^ and also investigated nerve synapse signaling (*cdk-5* and *unc-13* mutants),^44^^,^^45^^,^^46^ AMPK signaling (*aak-2* mutant),^47^^,^^48^ and mitochondrial translation regulation (*atf-4* mutant).^49^ We performed lifespan analysis of control worms and mutants of each of these regulators (Figures 4A–4F; Table S1). We found lifespan extension still present upon LDN treatment in *pmk-1(km25)* (Figure 4A), *cdk-5(ok626)* (Figure 4B), *atf-4(ok576)* (Figure 4C), *aak-2(ok524)* (Figure 4D), *unc-13(n2813)* (Figure 5E) mutant strains, which suggested that these pathways were not involved in LDN-induced lifespan extension. However, we observed no lifespan extension in *skn-1(zu67)* mutant strain (Figure 4F), demonstrating that the lifespan extension induced by LDN was dependent on SKN-1. As healthspan plays a major role in longevity, we also measured the mobility in *skn-1(zu67)* mutants. At day 8 of adulthood LDN could preserve mobility levels in N2 worms (Figure 4G) but could not preserve mobility levels in the *skn-1(zu67)* mutant worms (Figure 4H). Taken together, we conclude that the healthspan and lifespan extension observed with LDN were dependent on the activity of SKN-1.

### Low-dose naltrexone promotes the translocation of transcription factor SKN-1 from the cytosol to the nucleus and activates the oxidative stress response

SKN-1, the worm gene orthologous to NRF2 in mammals, is a major oxidative stress response regulator.^50^ During adult stages, SKN-1 accumulates in the intestinal nuclei and promotes longevity and innate immunity by inducing genes involved in the detoxification of ROS, such as gamma-glutamine cysteine synthetase *gcs-1*, glutathione S-transferases *gst-7* and *gst-4*, which play key roles in increasing oxidative stress resistance and extending lifespan.^50^^,^^51^^,^^52^^,^^53^^,^^54^ Because SKN-1 has been shown to be transcriptionally regulated by other compounds,^55^ we next asked if SKN-1 was also transcriptionally regulated by LDN. We therefore measured the mRNA expression of SKN-1 after LDN treatment. However, there was no difference between treating with or without LDN, suggesting an alternative regulation exists (Figure 5A).

It is known that oxidative stress induces SKN-1 to translocate from the cytoplasm to the nucleus in *C. elegans*.^56^ Therefore, we aimed to explore whether the effect of LDN was induced by the nuclear localization of SKN-1 in the intestine, using GFP-tagged marker strains. The results indicated that LDN treatment caused an accumulation of SKN-1 in the nuclei of the intestine, with nearly a 2-fold increase in the number of nuclear translocations compared to untreated, a change which approached levels similar to what we observed in our positive control (100mM paraquat) (Figure 5B; Table S3). To address whether the oxidative stress response resulting from LDN treatment could be regulated by SKN-1, we examined LDN-induced GFP expression in the *gst-4::GFP* and *sod-3::GFP* reporter strains while knocking down *skn-1*. Here, we found the LDN-induced oxidative response was significantly reduced after the knockdown of *skn-1* (Figures 5D, 5E, S2A, and S2B).

Recently, studies have shown that *skn-1* not only regulates the oxidative stress response but also influences immunity in *C. elegans*.^57^^,^^58^ Therefore, we next aimed to assess whether LDN’s effect on innate immune gene expression was also regulated by *skn-1*. Assessing transcript abundances of innate immune gene expression (including *C17H12.8, C32H11.4, F49F1.6, F56D6.2, K08D8.5,* and *M02F4.7*), we found the activation of these genes upon LDN treatment was partially abolished in the *skn-1(zu67)* mutant (Figure 5C). Taken together, these results suggest that both LDN’s activation of oxidative stress-induced genes, and activation of some of innate immunity genes, are dependent on the nuclear accumulation of SKN-1.

## Discussion

**Figure 6:**
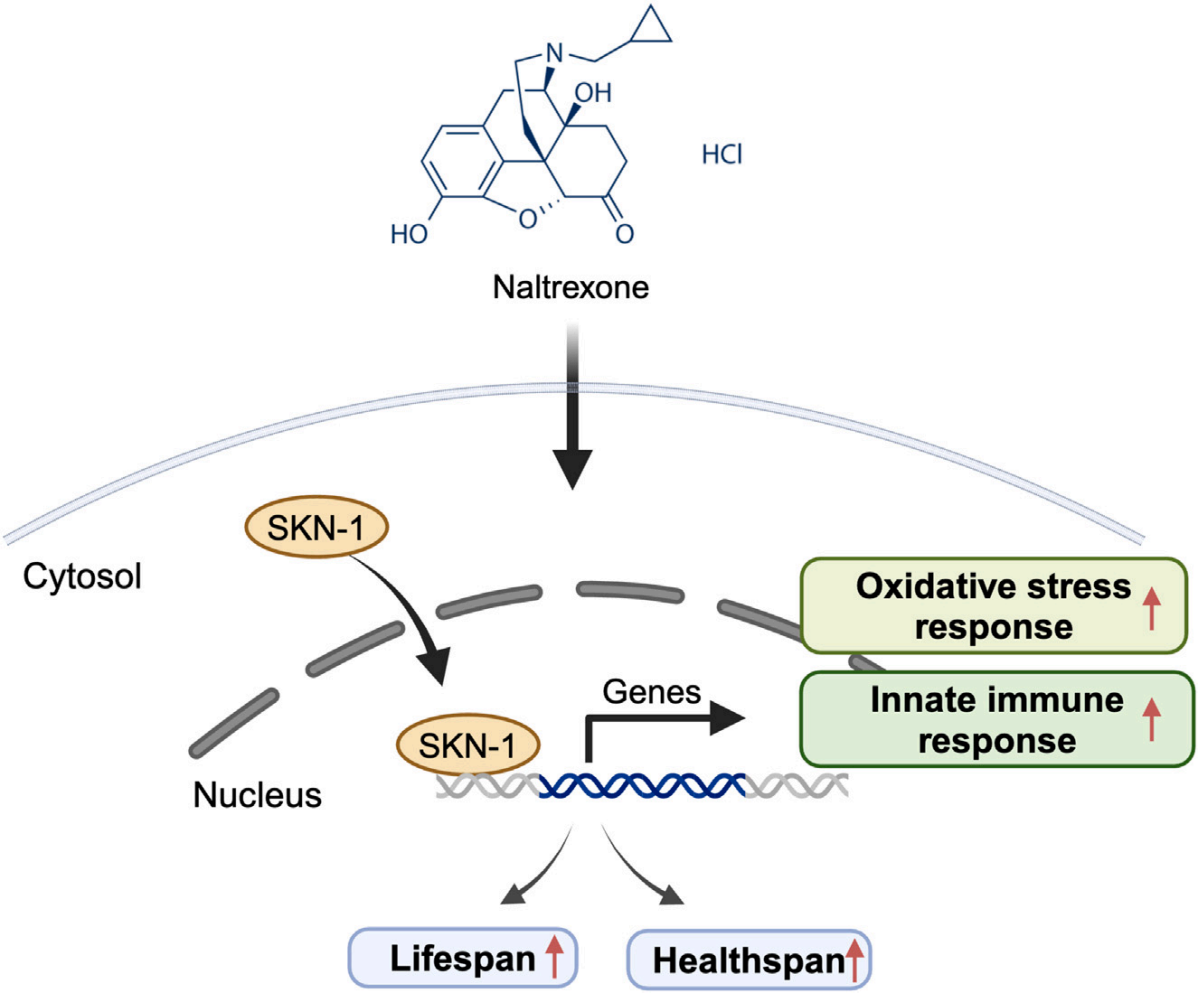
Schematic model of LDN’s mechanism LDN treatment results in the nuclear translocation of SKN-1, leading to the upregulation of the oxidative stress response, increased innate immunity gene transcription, and healthspan and lifespan extension.

In summary, our study uncovered a geroprotective role of LDN in promoting healthy aging in *C. elegans* (Figure 6). Specifically, we show that LDN treatment extended both healthspan and longevity by promoting the nuclear translocation of SKN-1, leading to the activation of the oxidative stress response and increased innate immunity gene expression. These findings suggested that LDN treatment may have therapeutic potential as an adjunct treatment modality for age-related diseases.

It is interesting to place these findings in the context of other work on LDN. LDN is proposed to be a potential treatment for cancers, autoimmune diseases, chronic pain, and mental health issues.^59^ Since many of these are related to inflammation, which is partly regulated by NRF2, it would be worth studying if the benefits of LDNs in these diseases come from NRF2 activation. The range of diseases for which LDN benefits is so diverse and large, that it may in fact be the case that LDN is affecting basic aging pathways in humans too. This possibility has been realized by others, and recently a clinical trial for LDN as a general treatment for slow aging has been proposed (NCT05307627). Our work suggests that LDN may indeed prove beneficial, and the mechanism rests on NRF2 activation. It would be interesting to study further NRF2 activity in LDN treated older individuals.

### Limitations and considerations of the study

A number of limitations accompany our findings that should be considered. For example, our metabolomics analysis was performed on whole-worm bulk samples and it cannot be ascertained what cells contributed to the changes observed. For example, having performed the metabolomics at the young adult stage, differences in egg content between treated and untreated worms may contribute to the differences observed. Another consideration pertains to the genetic epistasis analyses we performed (Figure 4). While these experiments revealed that lifespan extension was absent in the SKN-1 mutant strain, it could not fully exclude the possibility that other factors are also involved. This is since LDN could not reach the same lifespan extension in any of the mutant strains that was observed in the N2 strain (17.6%), or because mutant-specific genetic backgrounds may play a role.

Another consideration pertains to the activation of the innate immune response upon LDN treatment. Our work revealed that some, but not all, of our innate immune makers were induced upon LDN treatment (Figure 5C). Considering the low number of genes tested (*n* = 6), follow up experiments using RNAseq for a comprehensive assessment of the transcriptome would deepen our understanding of the extent to which LDN activates the innate immune response, as well as of what other pathways may be involved. Furthermore, the expression of the innate immunity genes we tested is downstream of PMK-1/MAPK signaling (Figures 3A and 3B). Given the role of PMK-1 in innate immunity regulation, it would be of interest to examine whether LDN-induced changes in innate immunity gene expression are abrogated in *pmk-1* mutants. This would help better understand the effects of LDN on innate immunity. Likewise, a more formal assessment of LDN’s ability to activate the immune response would involve pathogen assays and testing for improved pathogen resistance. Regardless of this outcome, however, our data argue against the involvement of PMK-1/MAPK-dependent innate immune regulation in the lifespan effects of LDN (Figure 4A).

Other considerations relate to the activation of the oxidative stress response upon LDN treatment. Similarly to the innate immune response, for the oxidative stress response, we report the induction of reporter genes (SOD-3 and GST-4) expression by LDN treatment and the lack of this induction upon SKN-1 knockdown. While this suggests the SKN-1-dependent activation of the oxidative stress response upon LDN treatment, it does not formally show an improved stress resistance. Therefore, assessing survival under conditions of oxidative stressors would be of interest to determine if LDN provides oxidative stress resistance. Furthermore, given the dependence of SKN-1 (a stress response activator) for lifespan and healthspan extension for LDN treatment, it is interesting to consider the possibility that naltrexone is working through hormesis.^60^ For example, naltrexone may even be causing oxidative stress itself. In a hormetic response, low doses stimulate adaptive stress responses, leading to beneficial effects on healthspan and lifespan, while a detrimental effect occurs at higher doses, with the treatment being essentially poisonous.^60^ Based on the doses we tested of naltrexone however, it is unlikely that a hormetic response is occurring, as our initial titration assay that identified the low dose (2.5μM) revealed that doses as high as 40x this amount (100μM) did not negatively influence worm healthspan. Furthermore, other researchers have used doses of Naltrexone on *C. elegans* in the millimolar range^61^ suggesting that toxicity is limited and therefore a hormetic response unlikely.

Of additional consideration, our work has been conducted entirely in *C. elegans* worms. While worms allow discerning mechanisms and mode of action of compounds more easily in the context of aging, it nonetheless suffer the drawback of being a simple, short-lived invertebrate, and the translation of findings to humans may differ. Furthermore, we have defined low-dose naltrexone in worms as 2.5μM, with higher doses being 50 and 100μM. We considered this to be a “low” dose because our previous work using other compounds often works with higher doses of drugs to achieve lifespan benefits, including 50 and 100μM.^16^^,^^19^ While the dose of 2.5μM was indeed optimal for lifespan extension, it may still be the case that translating this to humans does not match the same low dose used in LDN human studies. Additionally, while the lifespan extension from 2.5μM naltrexone treatment was robustly associated with lifespan extension in our work, the median lifespan extension of 17.6% is on the shorter side of the lifespan extension possible in worms. While some may view this as a limitation, other recent work from our team has demonstrated that the quantity of lifespan extension in worms from small molecule treatments is directly related to the probability of the small molecule causing large side effects in humans.^62^ Therefore, the lower, though significant lifespan extension we observe with LDN, paired with clear healthspan extension in our study, may in fact be the most optimal result when aiming to translate a longevity drug from worms to humans. Indeed, metformin—another repurposed drug candidate for human longevity greatly appreciated for its low-side-effect profile^63^ has had studies demonstrate median lifespan extensions in worms including 21.65%,^64^ 24.46%,^65^ a range from 6.3 to 57.3%,^66^ a range from 24.2 to 36.9%^67^ and 43.65%^68^ depending on the assay and study (percentages reported through DrugAge^69^). These lifespan extension percentages are also in the lower range of what is possible in worms (the largest effect reported in DrugAge^69^ is an extension of 75% from treatment with Thioflavin T under specific conditions^70^).

Therefore, the lifespan extension we observe for LDN treatment with a median of 17.6% may not be far off from what is required for the successful translation of a low-side-effect profile drug to humans.

Open questions remain following our findings. For example, while we demonstrate NRF2 dependency for LDN’s lifespan benefits, it is unclear if the direct downstream regulators of lifespan are related to oxidative stress, innate immunity, or both. Future work is necessary to clarify this. Whichever the case, what has emerged as a clear result, is the activation of NRF2 in LDN treatment as a requirement for lifespan and healthspan benefits. This is promising, considering that NRF2 has also been associated with lifespan extension in higher organisms.^71^^,^^72^ Taken together, our work suggests that LDN provides a useful strategy for counteracting the diseases of old age.

## STAR★Methods

### Key resources table

**Table:** 

REAGENT or RESOURCE	SOURCE	IDENTIFIER
**Bacterial and virus strains**		

*Escherichia coli*: OP50	*Caenorhabditis* Genetics Center	RRID:WB-STRAIN:OP50
*Escherichia coli*: HT115 (DE3)	*Caenorhabditis* Genetics Center	RRID:WB-STRAIN:HT115(DE3)
*skn-1* RNAi	Ahringer	T19E7.2

**Chemicals, peptides, and recombinant proteins**		

5-Fluorouracil (5-FU)	Sigma-Aldrich	Cat# F6627
Ampicillin sodium salt	Sigma-Aldrich	Cat# A9518
Carbenicillin disodium salt	Sigma-Aldrich	Cat# C1389
IPTG	AppliChem	Cat# A1008,0005
TriPure Isolation Reagent	Roche	Cat# 11667165001
Methanol (Optima™ LC/MS Grade)	Fisher Chemical	Cat# A454SK-4
Methyl tert-butyl ether (MTBE)	Sigma-Aldrich	Cat# 443808
18MΩ MilliQ water	Made in house	
Stainless metal bead (5mm diameter)	Qiagen	Cat# 69989
Acetonitrile (Optima™ LC/MS Grade)	Fisher Chemical	Cat# A955-4
Urea	Sigma-Aldrich	Cat# U5378
Tris(2-carboxyethyl) phosphine	Sigma-Aldrich	Cat# C4706
2-chloroacetamide	Sigma-Aldrich	Cat# C4706
Formic acid, Pierce™	Thermo Scientific	Cat# PI28905
Ammonium acetate (LiChropur™)	Sigma-Aldrich	Cat# 73594
Acetic acid	Sigma-Aldrich	Cat# 695092
Ammonium hydroxide	Sigma-Aldrich	Cat# 338818
2-Propanol (Optima™ LC/MS grade)	Fisher Chemical	Cat# A461212
Acetic acid	Sigma-Aldrich	Cat# 695092

**Critical commercial assays**		

NucleoSpin RNA, Mini kit for RNA purification	Macherey-Nagel	Cat# 740955.250
RNA using the Reverse Transcription Kit	Qiagen	Cat# 205314
LightCycler 480 SYBR Green I Master kit	Roche	Cat# 04887352001

**Deposited data**		

C. elegans metabolic data	This paper	Table S2, MTBLS7715; https://www.ebi.ac.uk/metabolights/MTBLS7715

**Experimental models: Organisms/strains**		

*C. elegans*: N2 Bristol	*Caenorhabditis* Genetics Center (CGC); https://cbs.umn.edu/cgc/home	RRID: WB-STRAIN: N2_(ancestral)
*C. elegans*: CF1038 [*daf-16(mu86) I*]	*Caenorhabditis* Genetics Center (CGC); https://cbs.umn.edu/cgc/home	RRID: WB-STRAIN:CF1038
*C elegans*: KU25 [*pmk-1(km25) IV*]	*Caenorhabditis* Genetics Center (CGC); https://cbs.umn.edu/cgc/home	RRID: WB-STRAIN:KU25
*C elegans*: RB814 [*cdk-5(ok626) III*]	*Caenorhabditis* Genetics Center (CGC); https://cbs.umn.edu/cgc/home	RRID: WB-STRAIN:RB814
*C. elegans*: RB790 [*atf-4(ok576) X*]	*Caenorhabditis* Genetics Center (CGC); https://cbs.umn.edu/cgc/home	RRID: WB-STRAIN: RB790
*C. elegans*: RB754[*aak-2(ok524) X*]	*Caenorhabditis* Genetics Center (CGC); https://cbs.umn.edu/cgc/home	RRID: WB-STRAIN:RB754
*C elegans*: MT8004 [*unc-13(n2813) I*]	*Caenorhabditis* Genetics Center (CGC); https://cbs.umn.edu/cgc/home	RRID: WB-STRAIN:MT8004
*C. elegans*: EU1 [*skn-1(zu67) IV*]	*Caenorhabditis* Genetics Center (CGC); https://cbs.umn.edu/cgc/home	RRID: WB-STRAIN: EU1
*C. elegans*: CL2166 [*dvIs19 [(pAF15)gst-4p::GFP::NLS] III*]	*Caenorhabditis* Genetics Center (CGC); https://cbs.umn.edu/cgc/home	RRID: WB-STRAIN:CL2166
*C elegans*: CF1553 [*muIs84 [(pAD76) sod-3p::GFP + rol-6(su1006)]*]	*Caenorhabditis* Genetics Center (CGC); https://cbs.umn.edu/cgc/home	RRID: WB-STRAIN:CF1553
*C. elegans*: LD1 [*dIs7 [skn-1b/c::GFP + rol-6(su1006)]*]	*Caenorhabditis* Genetics Center (CGC); https://cbs.umn.edu/cgc/home	RRID: WB-STRAIN:LD1

**Oligonucleotides**		

*cdc-42* Fw: TCGACAATTACGCCGTCACA	Sigma-Aldrich	N/A
*cdc-42* Rv: AGGCACCCATTTTTCTCGGA	Sigma-Aldrich	N/A
*rbd-1* Fw: AACTTGCCTAGCACCTGCAC	Sigma-Aldrich	N/A
*rbd-1* Rv: CATTCTTCAACCGTTAACTTTTTCG	Sigma-Aldrich	N/A
*pmp-3* Fw: GTCGAACGATGTGGTGGTCT	Sigma-Aldrich	N/A
*pmp-3* Rv: AGGCACCCATTTTTCTCGGA	Sigma-Aldrich	N/A
*C32H11.4* Fw: TCCTTTGAAATGCCGAGTCT	Sigma-Aldrich	N/A
*C32H11.4* Rv: GGCTTGTGAACCACATTTCC	Sigma-Aldrich	N/A
*F55G11.8* Fw: TCAAACAACCCACGAGAAAA	Sigma-Aldrich	N/A
*F55G11.8* Rv: AGCAAATCCTTCGTTGGAGA	Sigma-Aldrich	N/A
*F56D6.2* Fw: GGTGACAGTTCAAAGCCATGT	Sigma-Aldrich	N/A
*F56D6.2* Rv: TTCCAAAAATGCCCGAGTAG	Sigma-Aldrich	N/A
*M02F4.7* Fw: GGAACACTGGCTTCTGTTCAT	Sigma-Aldrich	N/A
*M02F4.7* Rv: CCAATATGAATTCGCTTGGAC	Sigma-Aldrich	N/A
*F49F1.6* Fw: CCATCAACTACGCCAAAGC	Sigma-Aldrich	N/A
*F49F1.6* Rv: TCCGGTGGATAGAAGGTGTT	Sigma-Aldrich	N/A
*skn-1* Fw: AGGCTCAACCTCAGAACATG	Sigma-Aldrich	N/A
*skn-1* Rv: AAATGAAATGAGACACGGCAAAGA	Sigma-Aldrich	N/A
*C17H12.8* Fw: TGTCATTTCAATGGAGGATATTGT	Sigma-Aldrich	N/A
*C17H12.8* Rv: TGATGGAGTTGGAGGATATTGA	Sigma-Aldrich	N/A
*K08D8.5* Fw: TACATTTTCACGTCCCCACA	Sigma-Aldrich	N/A
*K08D8.5* Rv: TGCATGTTCATTCGACTTCC	Sigma-Aldrich	N/A

**Software and algorithms**		

GraphPad Prism v9	GraphPad Software, Inc.	https://www.graphpad.com/scientificsoftware/prism/
R (version 4.2.2)	The R Foundation	https://www.r-project.org/
Adobe Illustrator 2023	Adobe	https://www.adobe.com/products/illustrator.html
MetaboAnalyst 3.0	MetaboAnalyst	https://www.metaboanalyst.ca/
openxlsx	openxlsx	https://cran.r-project.org/web/packages/openxlsx/index.html
dplyr	dplyr	https://CRAN.R-project.org/package=dplyr
xlsx	xlsx	https://CRAN.R-project.org/package=xlsx
ggplot2	ggplot2	https://cran.r-project.org/web/packages/ggplot2/index.html
pheatmap	pheatmap	https://cran.r-project.org/web/packages/pheatmap/

### Resource availability

#### Lead contact

Further information and requests for resource and reagents should be directed to and will be fulfilled by the Lead Contact, Georges E. Janssens (g.e.janssens@amsterdamumc.nl).

#### Materials availability

This study did not generate new materials.

#### Data and code availability

• The metabolomics data are available at MetaboLights MTBLS7715 (https://www.ebi.ac.uk/metabolights/MTBLS7715).
• This study did not generate any codes.
• Any additional information required to reanalyze the data reported in this work paper is available from the lead contact upon request.

### Experimental model and study participant details

#### *C. elegans* strains

*Caenorhabditis elegans* strains N2 Bristol, CF1038 [*daf-16(mu86)I*], KU25 [*pmk-1(km25)IV*], RB814 [*cdk-5(ok626)III*], RB790 [*atf-4(ok576)X*], RB754 [*aak-2(ok524)X*], MT8004 [*unc-13(n2813)I*]*,* EU-1 [*skn-1(zu67)IV*], CL2166 [*dvIs19 [(pAF15)gst-4p::GFP::NLS] III*], CF1553 [*muIs84((pAD76)sod-3p::GFP + rol-6(su1006))*], and LD1 [*ldIs7(skn-1b/c::GFP + rol-6(su1006))*] were obtained from Caenorhabditis Genetics Center (CGC). Worms were routinely maintained at 20°C with OP50 E. coli on nematode growth media (NGM) agar plates.

#### Bacterial strains

*Escherichia coli* HT115 (DE3) with the Empty Vector (EV) L4440 was obtained from the *Caenorhabditis* Genetics Center. Bacterial feeding RNAi experiments were performed as described.^73^ RNAi E. coli feeding clone used was *skn-1* (sequence: *T19E7.2*) derived from the Ahringer RNAi library.^74^ All these clones were confirmed by sequencing and knockdown efficiency was confirmed by qPCR. In all RNAi experiments described in this study, worms were subjected to RNAi bacteria from the time of hatching.

### Method details

#### Culture conditions

Naltrexone was obtained from Sigma Aldrich (N3136) and dissolved in water used for treatment and mixed in the NGM agar just before pouring. Worms were treated with compounds or water vehicles from the L4 stage onwards. Plates were changed at least once a week to ensure consistent exposure to the compound.

#### Mobility analysis

Gravid adult worms were age-synchronized using alkaline hypochlorite treatment and incubated in M9 buffer overnight. L1 stage worms were seeded to NGM plates. Worms were transferred to plates supplemented with compounds and 15 μM 5-fluorouracil (Sigma-Aldrich) at the L4 larval stage. All assays were performed at 20°C, and the L4 stage was counted as day 0 of life. At the stated day of adulthood, ∼50 worms were transferred to NGM plates without *E. coli* OP50, stimulated by tapping the plate, and immediately recorded for 200 cycles at room temperature using a Leica (Amsterdam, The Netherlands) M205 FA fluorescent microscope and Leica DFC 365 FX camera. Images were captured using Leica Application Suite X software, then processed with the wrMTrck plugin for ImageJ.^75^ Measurements of body size were also derived from these analyses. Data from wrMTrck were analyzed and visualized using RStudio. Statistical analysis compared conditions to their respective control with a one-way ANOVA corrected for multiple testing.

#### Lifespan measurement

Prior to experiments, all animals were maintained at a permissive temperature and grown for at least two generations in the presence of food to assure health. Lifespan analyses were conducted at 20°C. Synchronized L1 worms were fed with *E. coli* OP50, grown to the L4 stage, and then 100 worms were transferred to a new plate contains with 15 μM 5-fluorouracil (5-FU). L4 stage was counted as day 0 of life. Animals were counted every other day and scored as dead when they did not respond to the platinum wire pick. Worms that were missing, displaying internal egg hatching, losing vulva integrity, and burrowing into NGM agar were censored. In Figure 1D, all replicates treated with or without 2.5 μM naltrexone in N2 worms were merged to make a single lifespan curve. Survival plots, p values (Log-Rank), and proportional hazards were determined by using GraphPad Prism 9 software.

#### Confocal microscopy

Worms expressing SKN-1B/C::GFP were synchronized, grown and treated with compounds from L4 stage as described. To test SKN-1 nuclear localization, day 3 animals were collected and washed 3 times with M9 buffer for DAPI staining. After that animals were immobilized for 30 s in 40 mM tetramisole (Sigma Aldrich) in M9 buffer and mounted on 2% agarose pads. Nuclear localization of SKN-1B/C::GFP was visualized using a Leica DMI6000 inverted confocal microscope containing a 40×, 1.30 Oil CS2 objective lens and a Leica TCS SP8 SMD camera. Images were captured using Leica Application Suite X software. All samples were imaged at room temperature. Images of 20–30 worms per condition were taken over two independent experiments, and results were compiled from both replicates. The calculation of nuclear translocations was determined by randomly selecting 10 images from each condition and manually counting the number of nuclei with translocalization and the total number of nuclei (Table S3).

#### Fluorescent microscopy

CL2166 *[dvIs19((pAF15)gst-4p::GFP::NLS)]* and CF1553 *[muIs84((pAD76)sod-3p::GFP + rol-6(su1006))]* strains were grown in NGM plates either with or without the addition of NTX (2.5 μM), and seeded with E. coli OP50, HT115 and *skn-1* RNAi, until day 3 (∼96 h after L4 stage). Both vehicle and *skn-1*(RNAi) animals were stage-matched, worms were prepared for fluorescence microscopy, by preparing NGM plates without *E. coli* and adding a drop of tetramisole (40 mM). Worms were oriented using a picking tool. Images of vehicle and NTX-treated animals were using the same respective settings. Fluorescence exposure time was set to a value where vehicle-treated worms were barely visible and the same settings were used to analyze the NTX-treated worms. To show the results, the same level of brightness and contrast was applied to vehicle- and LDN-treated animals, with no impact on the fluorescence quantification of the images.

#### Total RNA isolation

N2 *C. elegans* from L4 stage were exposed to either vehicle (water) or 2.5 μM naltrexone (Sigma-Aldrich, USA). On adult day 3, nematodes were collected by washing off the plates with 3 times M9 buffer and 2 times water before being snap frozen in liquid nitrogen. For isolation of total mRNA, whole worms were homogenized with a 5 mm steel bead using a TissueLyser II (QIAGEN) for 5 min at frequency of 30 times/second. Cleanup of the RNA was performed according to RNeasy Mini Kit on the QIAcube per the manufacturer’s protocol (QIAGEN). Contaminating genomic DNA was removed using RNase-Free DNase (QIAGEN RNeasy Mini Kit on the QIAcube per the manufacturer’s protocol (Qiagen, California). RNA concentration and quality was measured by Nanodrop (Thermo Scientific; Breda, The Netherlands) and stored at −80°C until use.

#### Q-RTPCR

1 μg of extracted RNA was reverse transcribed into cDNA according to the instructions of the QuantiTect Reverse Transcription Kit (QIAGEN; Venlo, The Netherlands). Quantitative gene expression analysis was performed using the LightCyclerⓇ 480 SYBR Green I Master (Roche; Woerden, The Netherlands) and measured using the LightCyclerⓇ 480 Instrument II (Roche). Gene-specific primers were synthesized according to the sequences in Table S4. The N0 values of target genes were normalized to the geometric mean of reference genes *pmp-3* and *cdc-42*.

#### Metabolomics profiling

To evaluate changes of the metabolome in that with and without naltrexone-treated worms, whole body tissues of adult Day 3 worms were collected (treated from L4). Six replicates were prepared for each group, each of ∼2500 worms. Worm samples were then freeze-dried overnight.

In a 2 mL tube, the following amounts of internal standard dissolved in water were added to each sample of freeze-dried worms: adenosine-^15^N_5_-monophosphate (5 nmol), adenosine-15N5-triphosphate (5 nmol), D4-alanine (0.5 nmol), D7-arginine (0.5 nmol), D3-aspartic acid (0.5 nmol), D3-carnitine (0.5 nmol), D4-citric acid (0.5 nmol), 13C1-citrulline (0.5 nmol), 13C6-fructose-1,6-diphosphate (1 nmol), 13C2-glycine (5 nmol), guanosine-15N5-monophosphate (5 nmol), guanosine-15N5-triphosphate (5 nmol), 13C6-glucose (10 nmol), 13C6-glucose-6-phosphate (1 nmol), D3-glutamic acid (0.5 nmol), D5-glutamine (0.5 nmol), D5-glutathione (1 nmol), 13C6-isoleucine (0.5 nmol), D3-lactic acid (1 nmol), D3-leucine (0.5 nmol), D4-lysine (0.5 nmol), D3-methionine (0.5 nmol), D6-ornithine (0.5 nmol), D5-phenylalanine (0.5 nmol), D7-proline (0.5 nmol), 13C3-pyruvate (0.5 nmol), D3-serine (0.5 nmol), D6-succinic acid (0.5 nmol), D4-thymine (1 nmol), D5-tryptophan (0.5 nmol), D4-tyrosine (0.5 nmol), D8-valine (0.5 nmol). Subsequently, solvents were added to achieve a total volume of 500 μL methanol and 500 μL water. A 5 mm stainless steel bead was added and a Qiagen TissueLyser II was used to homogenize each sample, before the addition of 1 mL chloroform. After thorough mixing, samples were centrifuged for 10 min at 14.000 rpm. The polar top layer was transferred to a new 1.5 mL tube and dried using a vacuum concentrator at 60°C. Dried samples were reconstituted in 100 μL 6:4 (v/v) methanol:water.

Metabolites were analyzed using a Waters Acquity ultra-high performance liquid chromatography system coupled to a Bruker Impact II Ultra-High Resolution Qq-Time-Of-Flight mass spectrometer. Samples were kept at 12°C during analysis and 5 μL of each sample was injected. Chromatographic separation was achieved using a Merck Millipore SeQuant ZIC-cHILIC column (PEEK 100 × 2.1 mm, 3 μm particle size). Column temperature was held at 30°C. Mobile phase consisted of (A) 1:9 (v/v) acetonitrile:water and (B) 9:1 (v/v) acetonitrile:water, both containing 5 mmol/L ammonium acetate. Using a flow rate of 0.25 mL/min, the LC gradient consisted of: Dwell at 100% Solvent B, 0–2 min; Ramp to 54% Solvent B at 13.5 min; Ramp to 0% Solvent B at 13.51 min; Dwell at 0% Solvent B, 13.51–19 min; Ramp to 100% B at 19.01 min; Dwell at 100% Solvent B, 19.01–19.5 min. Equilibrate column by increasing flow rate to 0.4 mL/min at 100% B for 19.5–21 min. MS data were acquired using negative and positive ionization in full scan mode over the range of m/z 50–1200. Data were analyzed using Bruker TASQ software version 2021.1.2.452. All reported metabolite intensities were normalized to total protein content in samples, determined using a PierceTM BCA Protein Assay Kit, as well as to internal standards with comparable retention times and response in the MS. Metabolite identification has been based on a combination of accurate mass, (relative) retention times, ion mobility data and fragmentation spectra, compared to the analysis of a library of standards. Processed metabolomics data can be found in Table S2. Partial least-squares discriminant analysis (PLS-DA) was performed using mixOmics72 setting a variable of importance (VIP) score of greater than 1 as significant. Metabolite Set Enrichment Analysis (MSEA) was analyzed using MetaboAnalyst 3.0 software under the p-value < 0.05. The metabolomics data are available at MetaboLights MTBLS7715 (https://www.ebi.ac.uk/metabolights/MTBLS7715). The fully processed metabolomics can be found in Table S1.

#### Statistical analysis

All data presented are specified in the respective figure legends and results section. Statistical tests are indicated in the Figure legends. P values of less than 0.05 were considered significant. Statistical analyses were performed using the Prism 9 software (GraphPad Software, La Jolla, CA, USA) and R v4.2.2 as described in each respective STAR Methods section.

### Quantification and statistical analysis

No statistical methods were applied to pre-determine worm sample size. Comparison between more than two groups was assessed by using a One-way ANOVA test and two groups by using a student’s t-test. Prism 8 (GraphPad Software) was used for statistical analysis of all lifespans, qRT-PCR, and fluorescent microscopy experiments. All p<0.05 were significant. (∗∗∗∗*p*<0.0001; ∗∗∗*p*<0.001; ∗∗*p*<0.01; ∗*p*<0.05; n.s., not significant.) For lifespan comparison in worms was assessed by using the log-rank test. All experiments were done non-blinded.

## Supplemental information

Document S1. Figures S1 and S2 and Tables S1, S3, and S4

Table S2. C. elegans Metabolomics data, related to Figure 2
