# Supplementary material for: Low-dose naltrexone extends healthspan and lifespan in C. elegans via SKN-1 activation
Source: iScience. 2024 May 8;27(6):109949. doi: 10.1016/j.isci.2024.109949 (PMC11126937; doi:10.1016/j.isci.2024.109949)
Supplement: Document S1. Figures S1 and S2 and Tables S1, S3, and S4 [file mmc1.pdf]

iScience, Volume *27*

Supplemental information

Low-dose naltrexone extends healthspan and lifespan in *C. elegans* via SKN-1 activation

Weisha Li, Rebecca L. McIntyre, Bauke V. Schomakers, Rashmi Kamble, Anne H.G. Luesink, Michel van Weeghel, Riekelt H. Houtkooper, Arwen W. Gao, and Georges E. Janssens

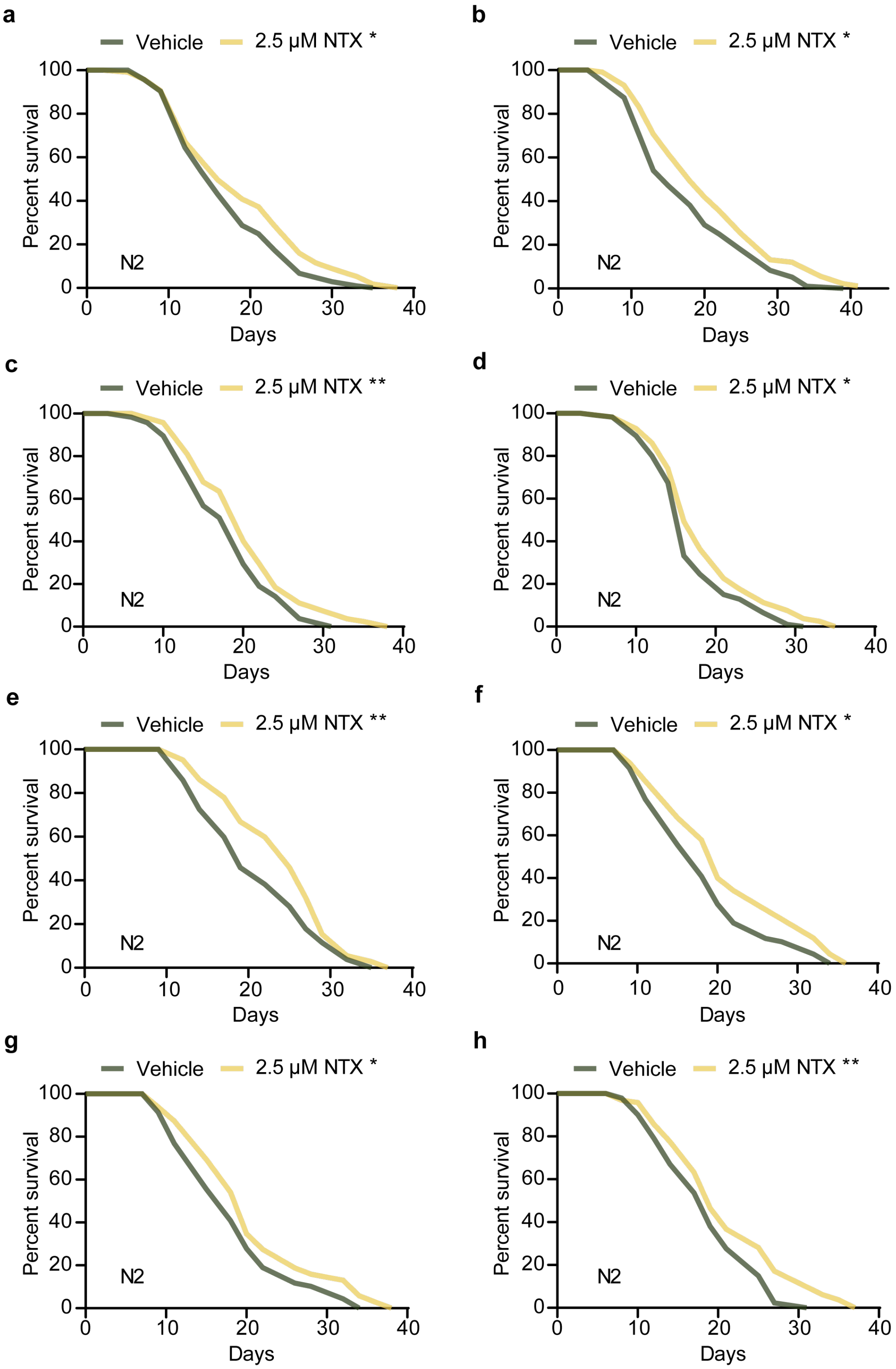

Figure S1. Impact of LDN on lifespan in individual experiments of N2 *C. elegans,* related to main text Figure 1D. (**a-h**) Independent lifespan assays of N2 worms treated with or without 2.5 μM NTX. Related to Figure 1d. The statistical analysis is performed by the log-rank test. **p <0.01, *p < 0.05.

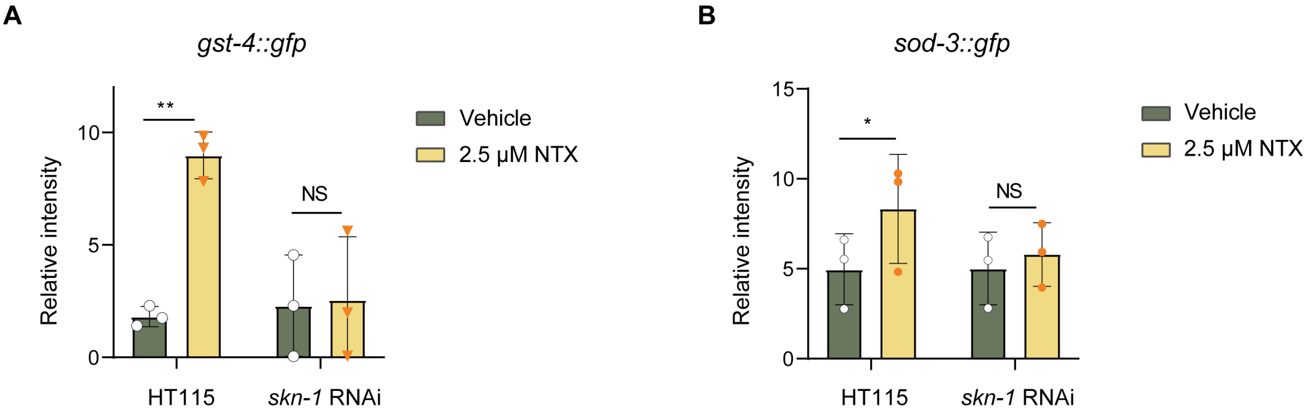

Figure S2. Quantification of GFP signaling on oxidative stress response reporters, related to main text Figure 5E and D. (**a**) Three independent tests for GST-4 signaling with or without LDN treatment. (**b**) Three independent tests for SOD-3 signaling with or without LDN treatment. Significance was determined using paired student's t-test (paired per independent replicate). $^{**}p < 0.01$, $^{*}p < 0.05$, NS: not significant.

Table S1: Lifespan Statistics, related to main text Figure 1, Figure 3 and Figure 4.

C. elegans strain	Treatment	Median lifespan (days)	% Change	Number animals (died/total)	P – value against control group
**Figure 1 Panel D**					
Wild Type (N2)*	water vehicle	17		732/880	
	2.5 μM naltrexone	20	17.65%	702/880	<0.0001
**Figure S1 Panel A**					
Wild Type (N2)*	water vehicle	16		108/120	
	2.5 μM naltrexone	16	0.00%	114/120	0.0288
**Figure S1 Panel B**					
Wild Type (N2)*	water vehicle	15		104/120	
	2.5 μM naltrexone	18	20.00%	102/120	0.0096
**Figure S1 Panel C**					
Wild Type (N2)*	water vehicle	20		109/120	
	2.5 μM naltrexone	20	0.00%	101/120	0.0097
**Figure S1 Panel D**					
Wild Type (N2)*	water vehicle	16		100/120	
	2.5 μM naltrexone	16	0.00%	90/120	0.0262
**Figure S1 Panel E**					
Wild Type (N2)*	water vehicle	19		80/100	
	2.5 μM naltrexone	25	31.58%	73/100	0.0124
**Figure S1 Panel F**					
Wild Type (N2)*	water vehicle	18		71/100	
	2.5 μM naltrexone	20	11.11%	71/100	0.0136
**Figure S1 Panel G**					
Wild Type (N2)*	water vehicle	18		71/100	
	2.5 μM naltrexone	20	11.11%	72/100	0.0498
**Figure S1 Panel H**					
Wild Type (N2)*	water vehicle	19		89/100	
	2.5 μM naltrexone	19	0.00%	84/100	0.0062
**Figure 1 Panel E**					
Wild Type (N2)*	water vehicle	20		109/120	
	50 μM naltrexone	20	0.00%	114/120	0.8371
Wild Type (N2)	water vehicle	18		71/100	
	50 μM naltrexone	20	11.11%	82/100	0.5641
Wild Type (N2)	water vehicle	19		89/100	
	50 μM naltrexone	19	0.00%	88/100	0.9860
**Figure 3 Panel F**					
*daf-16(mu86)**	water vehicle	14		109/120	
	2.5 μM naltrexone	16	14.30%	114/120	0.0040
daf-16(mu86)	water vehicle	17		78/100	
	2.5 μM naltrexone	17	0.00%	72/100	0.0412
**Figure 4 Panel A**					
*pmk-1(km25)**	water vehicle	19		79/100	

	2.5 µM naltrexone	19	0.00%	74/100	0.0011
pmk-1(km25)	water vehicle	20		69/80	
	2.5 µM naltrexone	22	10.00%	60/80	0.0024
**Figure 4 Panel B**					
*cdk-5(ok626)**	water vehicle	17		84/100	
	2.5 µM naltrexone	22	29.41%	76/100	0.0486
cdk-5(ok626)	water vehicle	19		85/100	
	2.5µM naltrexone	22	15.79%	79/100	0.0397
**Figure 4 Panel C**					
*atf-4(ok576)**	water vehicle	22		80/100	
	2.5 µM naltrexone	22	0.00%	80/100	0.0009
atf-4(ok576)	water vehicle	21		102/120	
	2.5 µM naltrexone	23	9.52%	103/120	0.0399
atf-4(ok576)	water vehicle	22		85/100	
	2.5 µM naltrexone	25	13.64%	73/100	0.0183
**Figure 4 Panel D**					
*aak-2(ok524)**	water vehicle	22		57/80	
	2.5 µM naltrexone	24	9.09%	50/80	0.0458
aak-2(ok524)	water vehicle	17		61/80	
	2.5µM naltrexone	19	11.76%	63/80	<0.0001
**Figure 4 Panel E**					
*unc-13(n2813)**	water vehicle	27		64/80	
	2.5 µM naltrexone	29	7.41%	58/80	0.0458
unc-13(n2813)	water vehicle	26		68/80	
	2.5 µM naltrexone	28	7.69%	66/80	0.0356
**Figure 4 Panel F**					
*skn-1(zu67)**	water vehicle	21		68/80	
	2.5 µM naltrexone	23	9.52%	66/80	0.4790
skn-1(zu67)	water vehicle	17		63/70	
	2.5 µM naltrexone	21	19.05%	65/70	0.8530
skn-1(zu67)	water vehicle	21		46/50	
	2.5 µM naltrexone	21	0.00%	45/50	0.9013

*Experiment represented in figure.
Note: statistics (P-value against control group) is determined using the entire lifespan curve (log-rank test), while the % Change represents the comparison of a single point in the lifespan curve (the median). Therefore, a % change may equal 0% if the populations reached 50% viable on the same day, while the lifespan curves are still significantly different.

Table S3: Quantification of the number of nuclear translocations, related to main text Figure 5B.

	Vehicle	LDN-treatment	100mM Paraquat
**Number of SKN-1cnuclear translocations (10 images, from 2 independent experiments)**	12	23	21
**Total nuclei observed (10 images)**	29	28	31

*A Chi-square test of independence was performed to assess the significance of the difference in the level of *skn-1* nuclear translocation between vehicle and LDN treatment, $p < 0.025$. No significance was observed between LDN treatment and the positive control of 100mM paraquat (Chi-square test).

Table S4: Primers used for *C. elegans* qPCR, related to the STAR Methods Q-RTPCR.

**Gene**	**Forward (sequence 5'→3')**	**Reverse (sequence 5'→3')**
**Reference gene**		
cdc-42	TCGACAATTACGCCGTCACA	AGGCACCCATTTTTCTCGGA
rbd-1	AACTTGCCTAGCACCTGCAC	CATTCTTCAACCGTTAACTTTTTCG
pmp-3	GTCGAACGATGTGGTGGTCT	AGGCACCCATTTTTCTCGGA
**Target genes**		
C32H11.4	TCCTTTGAAATGCCGAGTCT	GGCTTGTGAACCACATTTCC
F55G11.8	TCAAACAACCCACGAGAAAA	AGCAAATCCTTCGTTGGAGA
F56D6.2	GGTGACAGTTCAAAGCCATGT	TTCCAAAAATGCCCGAGTAG
M02F4.7	GGAACACTGGCTTCTGTTCAT	CCAATATGAATTCGCTTGGAC
F49F1.6	CCATCAACTACGCCAAAGC	TCCGGTGGATAGAAGGTGTT
skn-1	AGGCTCAACCTCAGAACATG	AAATGAAATGAGACACGGCAAAGA
C17H12.8	TGTCATTTCAATGGAGGATATT GT	TGATGGAGTTGGAGGATATTGA
K08D8.5	TACATTTTCACGTCCCCACA	TGCATGTTCATTCGACTTCC
